# Isolating *Linum usitatissimum* L. Nuclear DNA Enabled Assembling High-Quality Genome

**DOI:** 10.3390/ijms232113244

**Published:** 2022-10-31

**Authors:** Ekaterina M. Dvorianinova, Nadezhda L. Bolsheva, Elena N. Pushkova, Tatiana A. Rozhmina, Alexander A. Zhuchenko, Roman O. Novakovskiy, Liubov V. Povkhova, Elizaveta A. Sigova, Daiana A. Zhernova, Elena V. Borkhert, Dmitry N. Kaluzhny, Nataliya V. Melnikova, Alexey A. Dmitriev

**Affiliations:** 1 Engelhardt Institute of Molecular Biology, Russian Academy of Sciences, Moscow 119991, Russia; 2 Federal Research Center for Bast Fiber Crops, Torzhok 172002, Russia; 3 All-Russian Horticultural Institute for Breeding, Agrotechnology and Nursery, Moscow 115598, Russia; 4 Moscow Institute of Physics and Technology, Moscow 141701, Russia; 5 Faculty of Biology, Lomonosov Moscow State University, Moscow 119234, Russia

**Keywords:** flax, *Linum usitatissimum*, nuclei extraction, high-molecular-weight DNA, nanopore, high-quality genome

## Abstract

High-quality genome sequences help to elucidate the genetic basis of numerous biological processes and track species evolution. For flax (*Linum usitatissimum* L.)—a multifunctional crop, high-quality assemblies from Oxford Nanopore Technologies (ONT) data were unavailable, largely due to the difficulty of isolating pure high-molecular-weight DNA. This article proposes a scheme for gaining a contiguous *L. usitatissimum* assembly using Nanopore data. We developed a protocol for flax nuclei isolation with subsequent DNA extraction, which allows obtaining about 5 μg of pure high-molecular-weight DNA from 0.5 g of leaves. Such an amount of material can be collected even from a single plant and yields more than 30 Gb of ONT data in two MinION runs. We performed a comparative analysis of different genome assemblers and polishers on the gained data and obtained the final 447.1-Mb assembly of *L. usitatissimum* line 3896 genome using the Canu—Racon (two iterations)—Medaka combination. The genome comprised 1695 contigs and had an N50 of 6.2 Mb and a completeness of 93.8% of BUSCOs from eudicots_odb10. Our study highlights the impact of the chosen genome construction strategy on the resulting assembly parameters and its eligibility for future genomic studies.

## 1. Introduction

For centuries, *Linum usitatissimum* L. has been cultivated mainly for two major purposes—oil and fiber production [1,2,3,4]. Today, the agriculture is unique in its vast application range. First, flaxseed gained popularity because of its high omega-3, lignans, and fiber content [5]. Therefore, it is a health-beneficial supplement for people and animals [1,6]. Second, flax oil is a component of coatings, paints, and enamels. Due to the presence of unsaturated bonds, fatty acids form a layer on a coated surface, protecting it from possible damage [7]. Finally, flax fiber is yet another valuable primary product for manufacturing clothes, paper, and composite [7,8,9]. Thus, flax oil and fiber are products of continuing interest.

However, the raw material for flax oil and fiber production provides much space for advancement [10,11,12,13]. While wild relatives demonstrate a significant level of genetic diversity, domesticated flax forms are prone to undergoing the bottleneck effect in selected traits [14,15,16]. Therefore, strategies opposite to single trait selection can be adopted in breeding to reach the desired quality of the raw material [17]. In this regard, genetic and omics technologies hide enormous potential for creating improved cultivars because genomic features and their regulation determine all important agronomic characteristics of a plant [18,19]. A complete and contiguous genome sequence has the power to reveal important agronomic traits in crops. Thus, using two potato reference genomes and genome sequencing data on twelve landraces, Kyriakidou et al. observed increased copy numbers of genes involved in disease resistance, tolerance to abiotic stress, and vegetative growth and development in the sequenced genomes [20]. Another instance is the study by Saint-Oyant et al., who assembled a high-quality *Rosa chinensis* genome and used it to establish the location of genomic regions regulating ornamental traits [21]. In maize, a whole-genome sequence from Pacific Biosciences (PacBio) reads enabled Li et al. to detect SNPs and variations in a quality protein genotype [22]. Therefore, an available genome assembly is a versatile tool for studying key agricultural traits.

Next-generation platforms opened the high-throughput sequencing era. The technologies boosted genome sequencing in various plant species, simplified the screening of hundreds of individuals and lowered the overall sequencing cost (https://1001genomes.org/; accessed on 29 September 2022) [23,24,25]. However, the second-generation sequencing platforms have their own limitations along with obvious advantages. For example, sequencing with Illumina has no strict requirements for the input DNA quality and outputs precise reads. However, the resulting read length can be insufficient for long repeat resolution, while plant genomes comprise a large proportion of repetitive sequences [26,27,28,29]. Polyploid genome assembly with next-generation sequencing data is no less challenging because allele variants can be mistaken for duplicated regions [30]. Finally, assemblies from only short reads are highly fragmented [31].

Nonetheless, the quality of the utilized sequence directly affects the effectiveness of the analysis. Incomplete fragmented assemblies from short reads conceal the actual genome structure, and may misinform on the genetic elements’ presence. In contrast, third-generation sequencing platforms have become a game changer in genomics. As a general trend, the contiguity of the assembled genomes has increased with third-generation sequencing applications [31]. This parameter is crucial for complex plant genomes with a high content of repetitive elements. The ratio can reach nearly three-quarters of an assembly. For example, in *Ammopiptanthus nanus*, 74% of the sequenced genome consists of repetitive sequences [32]. Such genome regions are easier to assemble from long reads than the short ones, as was demonstrated in the study on *Spirodela polyrhiza* [33]. In the work on *Camellia sinensis*, long PacBio reads were effectively used along with the Illumina ones to construct a quality assembly with 64% of transposable elements [34]. Furthermore, besides improving de novo genome assemblies, the new sequencing data type substantially facilitates a profound analysis of the received genomic sequence. Thus, a long-read *Utricularia gibba* assembly enabled detecting whole-genome duplications and identifying more protein-coding sequences than a short-read one [35]. Another example is a study on *Brassica napus*. Oxford Nanopore Technologies (ONT) and PacBio data were used to detect structural variations involved in adaptation and disease resistance of the plant [36]. Thus, long-read sequencing has the capacity to advance plant genomics considerably.

Moreover, the scientific community shows a growing interest in receiving more complete genomes of a plant instead of using a reference assembly [37]. Because of structural variations, a single genome of a plant is an unreliable starting point for further genetic studies. Similarly, resequencing genomes with short-read data provides insufficient information. Conversely, third-generation platforms simplify and accelerate whole-genome studies, including the construction of a contiguous assembly. This benefit can be used in pan-genomic studies if a researcher chooses to obtain a pan-genome with de novo sequencing [38]. In addition, a contiguous assembly from long reads upgraded to the chromosome level is a solid platform for future genetic research [39,40].

Unfortunately, there is no universal recipe for constructing a high-quality assembly. The final outcome depends on the genome structure itself and the chosen sequencing platform. For *L. usitatissimum*, five genomes are available in the databases [41,42,43,44]. However, among the four chromosome-scale assemblies, only the genome of the cultivar YY5 is produced from long PacBio reads [41]. Another long-read based assembly is the contig-scale one, which we performed previously from ONT data for the cultivar Atlant [43]. ONT is an affordable option to assemble a complex plant genome because it produces genomic reads of a hypothetically unlimited length. In practice, reads up to 4.2 Mb in length were obtained (https://nanoporetech.com/about-us/news/blog-kilobases-whales-short-history-ultra-long-reads-and-high-throughput-genome; accessed on 29 September 2022). Nonetheless, for the cultivar Atlant, we failed to obtain a high fraction of long reads and assemble a genome with QUAST parameters comparable to those of the YY5 assembly. Reaching a desired N50 can be a daunting challenge as the ONT platform requires extra-pure high-molecular-weight DNA [45,46]. In this regard, cell-metabolite content is a primary aspect to take into consideration before DNA isolation. Another important factor is the integrity of the cellular DNA, which can be easily sheared during isolation, e.g., mechanically. For these reasons, various DNA extraction approaches exist.

The majority of protocols are based on the use of cetyltrimethyl ammonium bromide (CTAB) [45,47,48,49,50,51]. However, the initial step of these procedures focuses on cellular membrane lysis, including the nuclear one. This allows extranuclear metabolites to interact with DNA and significantly complicates its further purification, reducing the amount of DNA suitable for third-generation sequencing. Moreover, all the subsequent manipulations with DNA inevitably damage the molecule, breaking its extra-long fragments. To mitigate these effects, DNA can be extracted from the isolated nuclei [52]. Nuclear DNA can be released directly from the agarose-embedded nuclei, or precipitated with a CTAB-buffer after the nuclear membrane lysis, or extracted with a commercial kit [53,54,55]. The choice of the method is a matter of both plant object characteristics and available resources. Thus, to sequence *Rehmannia glutinosa* on the ONT platform, Ma et al. used a CTAB-based protocol [55]. For *Lolium perenne* L., Frei et al. employed a protocol including cell wall lysis with further DNA precipitation and purification on magnetic beads [53]. Driguez at el. developed a general scheme of sequencing plant genomes on the PacBio platform and tested it on seven taxonomically diverse plant species. However, the authors suggest that the applied column-based approach is suitable for ONT applications [56]. Therefore, a protocol for DNA extraction should be tested for suitability for a studied plant species each time.

For sequencing *L. usitatissimum* on the ONT platform, a protocol for pure high-molecular-weight DNA isolation was absent. Our previous research showed that extracting DNA according to CTAB-based methods leads to insufficient DNA quality [41]. Therefore, a large number of sequencing runs and huge amount of plant material were necessary to obtain quality genome assembly from ONT reads. Based on affordable techniques, our novel protocol yields pure high-molecular-weight flax DNA to gain a sufficient number of long ONT reads. The approach enables assembling a high-quality genome even of a single flax plant.

## 2. Results

Using the developed protocol for pure high-molecular-weight DNA isolation (described in the Materials and Methods section), half a gram of raw plant leaves yielded five micrograms of flax DNA with A260/280~1.8 and A260/230~1.9. The DNA concentrations measured using a spectrophotometer and fluorimeter varied by no more than 10%. The total amount of the obtained DNA was sufficient for two runs on the R9.4.1 MinION flow-cells. We received 30.6 Gb of raw genome sequences with an average read length of 14.1 kb (14.1 and 16.5 Gb with an N50 of 12.4 and 15.7 kb, respectively).

Raw *L. usitatissimum* reads were basecalled with Guppy and the dna_r9.4.1_450bps_sup algorithm. According to Guppy basecalling reports, ~56% of all the collected data passed the quality threshold of ten. Draft genomes were assembled from the merged data with Q ≥ 10 using the relevant and long-known assemblers—Canu, Flye, Miniasm, NextDenovo, Raven, Shasta, SMARTdenovo, and Wtdbg2. To identify the most accurate assembly, we relied on the BUSCO completeness and QUAST parameters, including the reference-based ones (annotated reference genome assembled from Illumina data—variety CDC Bethune, GCA_000224295.1, PRJNA68161) (Table 1).

Three key parameters were taken into account during the comparison between the produced assemblies. First, the resulting genome length should be close to the expected one. Second, a raw assembly is considered the most contiguous if it has the highest N50 and the lowest number of contigs and percentage of fragmented BUSCOs (benchmarking universal single-copy orthologs). Finally, the completeness of an assembly is reflected in the percentage of complete BUSCOs, covered reference genome fraction, and number of detected reference genomic features (genes, CDS, etc.). In our analysis, Canu and Flye (from Canu-corrected reads) assembled the most contiguous genome sequences, according to the N50 statistic (6.2 and 5.8 Mb, respectively). The raw genomes by Flye and Canu also had the highest number of complete reference features as well as bases aligned with the reference genome of CDC Bethune (GCA_000224295.1). However, the assembly by Flye was 16% smaller than could be expected (evaluated flax genome length ~400 Mb) [42]. The lowest number of contigs was characteristic of SMARTdenovo and NextDenovo assemblies, which also had acceptable N50 values—2.8 and 3.1 Mb, respectively. Nonetheless, these raw genomes still missed nearly 60% (SMARTdenovo) and 28% (NextDenovo) of the expected length. Probably, this effect can be attributed to the algorithm type implemented in SMARTdenovo and NextDenovo and inadequacy of the genome coverage for these assemblers. In terms of BUSCO completeness, the assemblies by Canu, Flye (from Canu-corrected reads), and Shasta had the highest percentages of complete ortholog sequences (93.2–93.8%). Therefore, the Canu assembler constructed the most contiguous 447 Mb-long genome sequence.

This assembly was used for further polishing with ONT data to improve accuracy using four well-known polishers (Medaka, NextPolish, Pepper, Racon) several times each and in different combinations (Table 2).

All the polishing tools use different approaches to eliminate assembly errors. NextPolish has the k-mer score chain and count modules for genome correction [58]. Medaka and Pepper both employ neural networks to rectify mismatches and indels (https://github.com/nanoporetech/medaka; accessed on 29 September 2022) [59]. Racon uses the partial order alignment graph to refine raw assemblies [60]. However, systematic errors remain after the polisher and should be corrected with another instrument, e.g., Medaka. As shown in Table 2, according to QUAST and BUSCO statistics, all the used tools increased the accuracy of the assembly. Two rounds of Pepper resulted in the highest achieved BUSCO completeness and number of detected reference genomic features, as well as the lowest normalized number of mismatches and indels. Racon ×2 also showed good results, but they were inferior to those of Pepper ×2. However, Racon and Pepper handled the assembly size dissimilarly. The first iteration of Pepper’s polishing cut the total assembly length by 5%, and the second one—by 3%. In contrast, after Racon (both ×1 and ×2), the parameter values insignificantly fluctuated around 447 Mb. NextPolish showed the worst results. After Medaka, the assembly size slightly increased in both first and second polishing rounds. This tool significantly cut the relative numbers of mismatches and indels and improved the number of reference genomic features. Since Medaka was tailored to correct systematic errors, it was also tested in combination with Racon and Pepper. Compared to the Canu—Racon—Medaka combination, Canu—Pepper—Medaka resulted in more detected reference features and less mismatches and indels per 100 kb but significantly reduced the assembled genome length. Similarly, polishing the raw assembly with Pepper twice and Medaka once provided the optimal QUAST and BUSCO statistics, except that the length was reduced by 36 Mb. Racon ×2—Medaka was the second best polishing scheme among the other tested ones and kept most of the assembled genome. Thus, we considered the Canu assembly polished using Racon twice and Medaka once the most contiguous and accurate.

Next, we compared the QUAST and BUSCO parameters between the available flax genome assemblies (taken at the contig level), including the obtained 3896 one (Table 3).

The produced 3896 assembly had the second lowest number of contigs (1695) and the second highest N50 (6.2 Mb) among all the analyzed assemblies. Compared to the Atlant genome statistics, obtained by us earlier, the achieved QUAST parameters for line 3896 substantially improved. However, the achieved BUSCO completeness (93.8%) failed to exceed that of the Atlant assembly, likely because the Atlant assembly was additionally polished with Illumina reads. To study the repeat content, we used LTR_retriever [61]. Approximately half of line 3896 and YY5 genomes consisted of interspersed repeats (~49 and 50%, respectively). Meanwhile, the Atlant genome had 4–5% lower interspersed repeats content. For *L. bienne*, CDC Bethune, Heiya 15, and longya, the parameter varied in the range ~28–36%.

## 3. Discussion

Long-read sequencing technologies revolutionized plant genomics both in terms of study frameworks and methodology [40,62]. Long reads enable researchers to assemble large plant genomes de novo with improved contiguity, compared to the shotgun sequencing approach [63,64]. However, the third-generation sequencing technologies have their own limitations. Thus, although the Oxford Nanopore Technologies platform is unique in producing extremely long reads, the resulting data contains numerous errors. As time passes, sequencing technologies evolve. In May 2021, ONT announced new chemistry and flow-cells allowing one to achieve a better data quality and produce assemblies with a better QV (https://www.keygene.com/news-events/fast-contiguous-and-accurate-arabidopsis-col-0and-tomato-heinz-1706-genome-assembly-thanks-to-new-chemistry-nano-pores-and-plant-trained-basecaller/; accessed on 29 September 2022).

Nevertheless, several requirements for DNA quality are currently unavoidable. First, a high impurity concentration in a DNA sample drastically reduces the lifetime of the flow-cell pores leading to low data output. Therefore, more plant material and consumables are required to obtain a high-quality genome assembly. At the same time, plant cells are rich in polysaccharides inhibiting effective sequencing. Second, the higher the sheared DNA fraction, the more fragmented the assembled sequence is. Conversely, even a seemingly insignificant percentage of long reads leads to positive changes in assembly contiguity [65]. To obtain extremely pure and long DNA stretches, the protocol for DNA extraction should be adapted for the studied plant object. Currently developed methods for plant DNA isolation vary in labor-intensiveness and suit different needs. DNA can be pooled from enzymatically digested cells with magnetic beads, or isolated according to the CTAB method and additionally purified on both columns and beads, or released from the obtained plant nuclei [54,66,67,68,69,70]. The nuclei isolation approach has two powerful advantages for sequencing DNA on the ONT platform. The isolated nuclei can be efficiently washed from contaminating plant cell metabolites. In addition, the nuclear membrane preserves high-molecular-weight DNA, keeping the washing procedure safe.

In this study, we aimed to create a protocol for pure high-molecular-weight DNA extraction from flax nuclei. Previously, we employed DNA precipitation with a CTAB buffer for isolation of the cultivar Atlant DNA for sequencing on the ONT platform [43]. Despite the acceptable spectrophotometric values and close concentration values evaluated on Nanodrop and Qubit, the DNA quality was still inadequate to produce a really high amount of the sequencing data. We obtained 8.4 Gb of raw sequences with an average fragment length (N50) of 12 kb in one MinION run. In contrast, the current protocol allowed us to sequence about twice the amount of the earlier received data per run (1.8-fold increase on average). This increase indicates that the DNA purity improved significantly due to several factors. First, extracting from the washed nuclei allowed us to eliminate a considerable amount of contaminants at the washing step. Second, to reduce the initial impurity concentration and alleviate the washing procedure, we kept flax plants for 1 week in the dark. This step is conventionally used to metabolize polysaccharide content [71,72].

Despite the increased data amount, the N50 parameter of the received ONT reads was lower than could be expected. Conventionally, commercial kits are used to eliminate short DNA fragments and, therefore, increase N50 [36,46]. In this study, we assumed that a large fraction of long fragments could be obtained without the elimination procedure as the applied extraction method should have preserved long DNA fragments. Indeed, we received DNA fragments of ~200–1000 kb. Although the majority of the extra-long reads were filtered out during basecalling (average read quality of more than ten), 14% remained (up to 537 kb) and aligned with contigs from the final assembly. Therefore, manipulations with DNA during library preparation were unable to break super-long DNA stretches. Still, the resulting read length distribution was skewed to the short-read range. To improve N50 values in further work, the remaining short reads should be eliminated, e.g., using a Short Read Eliminator kit (Circulomics, Baltimore, MD, USA).

On obtaining and sequencing pure high-molecular-weight DNA, we designed a scheme for building the genome of *L. usitatissimum* line 3896. To construct an optimal draft genome, we tested a range of assembly tools (Table 1) as the software performs differently depending on the gathered data amount and quality, as well as genome length and complexity. According to our previous research, Canu—an overlap layout consensus algorithm—is the most advantageous software for a large volume of sequencing data [43,65,73,74]. Probably, it is the read-correction stage included in the pipeline that positively contributes to assembly contiguity. Nevertheless, the tool application is limited, since running all pipeline stages requires the highest number of CPU-hours. To surmount this obstacle but keep the advantage of an error-correction step, we used a more rapid assembler—Flye, to construct a genome from the Canu-corrected reads. Flye was also run on the uncorrected reads. The obtained assembly parameters expectedly improved for an assembly from the Canu-corrected reads compared to that from the uncorrected data. We observed a dramatic increase in the N50 value (from 0.3 to 5.8 Mb) and a decrease in the total number of contigs (from 7720 to 1571) while the assembly size was almost the same. BUSCO completeness and the percentage of detected reference genomic features also rose, indicating improvement in the assembly accuracy. However, the total genome length failed to reach the expected 400 Mb. The same was true for the other used assemblers—Miniasm, NextDenovo, Raven, Shasta, SMARTdenovo, Wtdbg2. Except for Shasta, assemblies produced with these tools also had the lowest BUSCO values (21.7–83.3%), while the assemblies by Shasta, Flye, and Canu had the highest ones. The high completeness values and insufficient genome length indicated that a considerable portion of the genome sequences was absent, but the majority of genetic features remained. The missing sequences could be repetitive.

Notably, the chosen sequencing technology and plant genotype contribute to the observed repeat content. Sa et al. sequenced the genome of the variety YY5 on a PacBio instrument and observed that ~55% of the resulting ~450 Mb-assembly consisted of repetitive elements, involving retro- and DNA transposons [41]. The authors also reannotated repeats from the assembly (GCA_000224295.2) of the flax variety CDC Bethune sequenced on the Illumina platform. The repeats occupied ~29% of the genome, while its size reached 316 Mb. In this study, the calculated total interspersed repeats content is ~49% for line 3896, ~50% for the YY5 genome, and ~28% for the CDC Bethune v.2 assembly. The high proportion of repetitive elements could explain the difference in genome sizes of the sequenced varieties.

However, the obtained genome length hinges on the chosen genome sequencing strategy. Building the first version of the CDC Bethune genome, Wang et al. measured the haploid genome size using flow cytometry. The established length was 373 Mb [75]. Thus, the second version of the obtained CDC Bethune assembly was still 15% smaller than could be expected (316 vs. 373 Mb). Although the genome was covered 94 times, the total ungapped length was ~270 Mb (https://www.ncbi.nlm.nih.gov/assembly/GCA_000224295.2; accessed on 29 September 2022). As the CDC Bethune assembly was constructed from highly accurate short Illumina reads, the technology imposes limitations on assembly contiguity. Conversely, both the YY5 assembly and the Canu-assembled genome in this study were constructed from third-generation sequencing data. PacBio and ONT reads cover longer stretches of a genome, leave considerably fewer gaps, and resolve repeats exceeding the maximum Illumina read length.

In addition to substantial repeat content, the flax genome contains a considerable fraction of duplicated genetic elements. We computed and compared percentages of conservative duplicated orthologs (BUSCOs) in the available *L. usitatissimum* and *L. bienne* assemblies (Table 3). The wild flax (*L. bienne*) assembly possesses the lowest number of duplicated BUSCOs of all the published genomes—50.4%, and the *L. usitatissimum* cultivar Atlant—the highest—63.4% [43,44]. In this work, genome assemblies by Canu, Flye, and Shasta had proximate numbers of duplicated orthologs (57.9–61.8%). The resulting values agree with a probable history of *L. usitatissimum* genome origin—the species probably originated from the hybridisation of two *Linum* species with diploidisation of the hybrid genome [76]. Therefore, the differences in genome lengths of the CDC Bethune, YY5, and line 3896 are attributed to other structures than duplicated coding sequences, e.g., the repetitive DNA content as we observed in this study.

Interestingly, the difference between duplicated BUSCO content was observed not only for different assembly tools, but polishing software (Table 2). To improve the quality of the draft genome, we chose and polished the assembly by Canu due to its optimal quality statistics combination (total length, N50, reference genomic features, and BUSCO completeness). Based on neural networks operation, Medaka and Pepper made the most changes in the duplicated BUSCOs ratio. Generally, all tested polishers increased the percentage of duplicated orthologs, pointing at the improvement in genome accuracy.

In terms of other parameters, polishing tools demonstrated variable performance. Regarding BUSCO completeness, detected reference genomic features, and mismatches/indels ratio, two rounds of Pepper outperformed other tools in a single-tool competition. However, it reduced the assembly size from 447 to 411 Mb. Nevertheless, the number of duplicated BUSCOs and BUSCO completeness changed in the opposite way. Therefore, although Pepper was developed as a haploid genome polisher, it could distinguish between gene duplicates. Thus, the reduction in size might have resulted from merging low-complexity regions. Coupling two iterations of the tool with Medaka resulted in the most prominent decrease in relative mismatches/indels numbers. Unlike Pepper, Racon corrected fewer mismatches and indels, but allowed reaching the same BUSCO completeness and kept the expected genome size nearly the same. In the end, Canu—Racon ×2—Medaka proved to be an optimal polishing scheme. However, the assembly BUSCO completeness was 93.8%. It can probably be improved with further polishing with highly accurate Illumina data.

**Figure 1:**
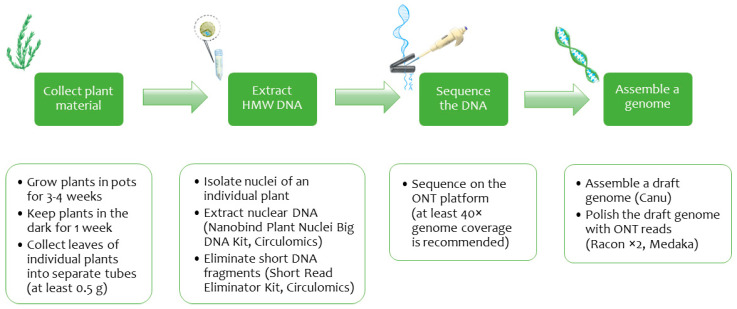
Scheme of obtaining a high-quality flax genome assembly from ONT reads.

In the current study, we developed a protocol for high-molecular-weight DNA isolation from flax nuclei of even an individual plant, sequenced the extracted nuclear DNA on the ONT platform, and assembled a contiguous genome (Figure 1). The scheme offers several huge benefits. First, sequencing a single plant genome has the merit of separating information on haplotypes, homozygous and heterozygous individuals. Therefore, the gained knowledge can find practical application in molecular research of such phenomena as heterosis [77]. Second, the employed DNA isolation method allowed us to receive an adequate long reads volume and assemble a contiguous *L. usitatissimum* genome of line 3896. A contiguous genome assembly is a firm foundation for a future complete genome. For example, a chromosome *Cymbidium sinense* assembly based on the ONT data allowed Yang et al. to study chromosome syntenies and reveal a whole-genome duplication [78]. Finally, ONT is still the leader in sequencing read length and cost-efficiency. Besides, ONT data facilitates studying nucleotide modifications as a bonus at the expense of genome sequencing [79]. In light of these opportunities, the developed protocol provides a powerful impetus to sequence new flax genomes de novo. From a methodological perspective, our approach for gaining a high-quality genome highlights the significance of correctly choosing plant material and DNA isolation technique, as well as designing an assembly scheme.

However, the currently obtained data still have room for improvement. The assembled line 3896 genome can be elevated to a chromosome level. Since no complete chromosome-scale genome can be currently assembled using a single sequencing technique, a variety of additional methods should be considered. Thus, combining different data types became a new paradigm in resequencing and de novo sequencing [36,79,80,81,82]. Assembling a genome from ONT and PacBio reads simultaneously allowed researchers to delve deep into genome structure, track rearrangements, and detect more transposable elements [36,79,83]. Other options are the use of optical maps and Hi-C scaffolding, which successfully link contigs to chromosomes The received genomes become powerful sources of genetic information [62,64,84]. Therefore, the received *L. usitatissimum* genome can be upgraded to a chromosome scale and used in comprehensive structure analyses.

## 4. Materials and Methods

### 4.1. Growing Plant Material

Seeds of *L. usitatissimum* line 3896 (highly resistant to *Fusarium* wilt (*Fu4* gene), rust, and neutral pH; late-maturing; high yield of linseed (in the Central Non-Black Earth region—1600 kg/ha); linseed fat content—40.5%) were provided by the Institute for Flax (Torzhok, Russia) [85]. The material was sterilized in 1% NaClO solution for 5 min, germinated on sterile petri dishes, and then planted in sterile soil. To minimize the content of metabolites, a 3–4 week-old plant was covered with dark cloth to prevent exposure to light. After 1 week of growth in the dark, plant leaves were collected, weighed, immediately frozen in liquid nitrogen, and stored at −70 °C until nuclei isolation.

### 4.2. Nuclei Isolation and DNA Extraction

Nuclei isolation was performed according to the steps 1–8 of the LN2 Plant Tissue Protocol (NUC-LNP-001, Circulomics, Baltimore, MD, USA) with modifications. Four buffers were prepared ahead of nuclei isolation: 10× HB (100 mM Trizma base (Sigma-Aldrich, St. Louis, MO, USA), 800 mM KCl (Scharlab, Barcelona, Spain), 100 mM EDTA (Promega, Madison, WI, USA))—adjusted to a pH of 9.0–9.4, 1× HB (10 mM Trizma base, 80 mM KCl, 10 mM EDTA, 0.5 M sucrose (Sigma-Aldrich)), TSB (Triton X-100 20% (*v/v*) (Sigma-Aldrich), 10 mM Trizma base, 80 mM KCl, 10 mM EDTA, 0.5 M sucrose), and NIB (10 mM Trizma base, 80 mM KCl, 10 mM EDTA, 0.5 M sucrose, Triton X-100 20% (*v/v*), 2 mM PVP K15 (PanReac AppliChem, Darmstadt, Germany), 1 mM spermine (Sigma-Aldrich), 1 mM spermidine (Sigma-Aldrich)). TSB was prepared from 1× HB and NIB—from 1× HB and TSB. Spermine, spermidine, and 375 µL of β-mercaptoethanol (Bio-Rad, Hercules, CA, USA) were added to the NIB right before use. The buffer was ice-cooled before nuclei isolation. The amount of the input material was reduced to ~0.5 g per 30 mL of the NIB buffer. Plant material was ground in liquid nitrogen to a flour-like powder, transferred into 30 mL of the ice-cooled NIB in a conical tube, and pipetted to break lumps. The tube was placed on ice and mixed end-over-end at 150 rpm for 15 min (PSU-10i orbital shaker, BioSan, Riga, Latvia). Then, cell lysate was consecutively filtered through 100 and 40 µM strainers (Corning, Corning, NY, USA) to remove debris. The filtrate was centrifuged at 4 °C at 60× *g* for 2 min for additional purification from remaining debris. The resulting supernatant was transferred to a clean conical tube and centrifuged for 20 min at 3220× *g* at 4 °C. The liquid fraction was decanted, and the formed pellet was resuspended in 3 mL of the NIB using a paint brush. Then, 30 mL of the NIB was added and mixed with the pellet. The mixture was centrifuged for 2 min at 4 °C at 60× *g*. The supernatant was placed in a clean conical tube and centrifuged for 10 min at 3220× *g* at 4 °C. The liquid fraction was decanted, and the pellet was washed 4 times: the precipitated fraction was resuspended, adjusted to a volume of 15 mL with NIB, and centrifuged for 10 min at 3220× *g* at 4 °C. After the last wash, the formed pellet was resuspended in 4 mL of the NIB and divided into two portions.

To isolate nuclei, density gradient centrifugation was employed. Density gradient comprised two layers. The upper layer mixture was prepared from 1.04 mL of iodixanol density gradient (10 mM Trizma base, 10 mM EDTA pH = 8, 80 mM KCl, 50% idoixanol (Sigma-Aldrich)) and 0.96 mL of 1× HB buffer. The lower layer mixture was obtained by adding 1.44 mL of iodixanol density gradient to 0.56 mL of HB. Using a wide-bore tip, 2 mL of the nuclei-containing suspension was layered on top of the gradient in a 15 mL conical tube. The remaining 2 mL of the nuclei suspension was placed in another tube containing a density gradient of the same composition. The tubes were centrifuged for 40 min at 3220× *g* at 4 °C. On nuclei band forming, 2 mL of the upper buffer layer was removed from each tube. For better purification, the nuclei band was pipetted to a width of 1 cm with a wide-bore tip. The tubes were centrifuged for 20 min at the same parameters. The upper buffer layer was removed, and the nuclei band was collected from each tube. The bands were combined and resuspended in 14 mL of the NIB. The suspension was centrifuged for 10 min at 2500× *g* at 4 °C. Then, the wash step with the NIB was repeated as described in the previous paragraph: supernatant was discarded, the nuclei were resuspended in 14 mL of the NIB, the mixture was centrifuged for 10 min at 3220× *g* at 4 °C. Supernatant was discarded, and the nuclei pellet was resuspended in 1 mL of the 1× HB buffer. The suspension was transferred to a 1.5 mL LoBind tube (Eppendorf, Hamburg, Germany) and centrifuged for 5 min at 7000× *g* at 4 °C. Liquid was discarded, and the nuclei pellet was used immediately for DNA extraction or stored at −70 °C up to one week.

The DNA was extracted from the isolated nuclei according to the Nanobind Plant Nuclei Big DNA Kit (Circulomics) protocol with minor modifications. On adding Proteinase K, the sample was vortexed and thoroughly mixed with a pipette tip due to its high viscosity. The step of 10 min incubation with RNAse A was included. On adding 80 µL of the PL1 buffer, the sample was vortexed for 1 s five times. During the incubation with the PL1 buffer, the sample was additionally vortexed for 1 s five times every 15 min. During the DNA precipitation step with the PW1 buffer, the tube was incubated for 15 min before placing it on a magnetic rack. To increase DNA yield, the nanobind disk was incubated with EB at RT overnight instead of 10 min. The quality of the obtained DNA was assessed with a Qubit fluorimeter, a Nanodrop spectrophotometer, and gel pulse-electrophoresis. The proximity of spectrophotometric and fluorometric concentration values served as a DNA purity indicator in addition to A260/280 and A260/230 ratios.

### 4.3. ONT Library Preparation and Sequencing

A DNA library was prepared using the SQK-LSK109 Ligation Sequencing Kit (ONT, Oxford, UK) for 1D genomic DNA sequencing. Minor modifications were introduced to the recommended protocol by increasing the incubation time to 20 min at 20 °C at the step of the DNA recovery and to 60 min at the ligation step. Sequencing was performed on the MinION instrument with a FLO-MIN-106D R9.4.1 flow-cell.

### 4.4. Genome Assembly

The fastq reads were derived from MinION fast5 files using Guppy 5.0.11 with the super accuracy flip-flop algorithm (dna_r9.4.1_450bps_sup.cfg). The adapter sequences were removed with Porechop (https://github.com/rrwick/Porechop; accessed on 29 September 2022). Raw genomes were assembled with Canu 2.2 (genomeSize = 400 m), Flye 2.9 (two assemblies—the first was generated from basecalled reads without adapters (flags: ‘--genome-size 400 m’, ‘--nano-raw’), and the second was produced from corrected reads output by Canu during assembly (flags: ‘--genome-size 400 m’, ‘--nano-corr’)), Miniasm 0.3, NextDenovo 2.5.0, Raven 1.8.1, Shasta 0.10.0, SMARTdenovo 0.1, Wtdbg2 2.5 (https://github.com/Nextomics/NextDenovo; accessed on 29 September 2022) [86,87,88,89,90,91,92]. As Canu assembled an optimal raw genome, the sequence was further used for polishing with Medaka 1.5.0 (-m r941_min_sup_g507), NextPolish 1.4.0, Pepper 0.1.1, Racon 1.4.10 (-m 8 -x -6 -g -8 -w 500) (https://github.com/nanoporetech/medaka; accessed on 29 September 2022) [58,59,60]. The quality of the resulting assembly was evaluated with QUAST statistics (QUAST 5.0.2, CDC Bethune v.1 as a reference—https://www.ncbi.nlm.nih.gov/assembly/GCA_000224295.1 (accessed on 29 September 2022), --fragmented option was used) and the presence of BUSCOs (BUSCO 4.1.2, eudicots_odb10) [57,93].

To compare the final line 3896 assembly with the available *L. usitatissimum* and *L. bienne* assemblies, the genomes were downloaded from NCBI and zenodo.org: Atlant (GCA_014858635.1), CDC Bethune v.1 and v.2 (GCA_000224295.1, GCA_000224295.2), longya 10 (GCA_010665275.2), Heiya 14 (GCA_010665265.1), YY5 v.2 (https://zenodo.org/record/4872894; accessed on 29 September 2022), and *L. bienne* 15003 (GCA_010665285.1). For the downloaded assemblies, QUAST statistics were taken from the NCBI and zenodo.org assembly descriptions. BUSCO statistics were calculated using the eudicots_odb10 dataset.

To calculate repeat content, we used LTR_retriever 2.9.0 including the BuildDatabase, RepeatModeler, and RepeatMasker modules. BuildDatabase was run on a given genome with default parameters [61]. Then, the created database was submitted to RepeatModeler with the “-engine ncbi” option. The output library (“consensi.fa.classified” file) was used as a RepeatMasker input (default parameters).

## 5. Conclusions

A successful protocol for plant DNA isolation significantly accelerates genomic studies if it requires low input of biological material and provides pure high-molecular-weight DNA. Such an approach should allow sequencing and assembling even a single-plant genome. In the current study, we developed a flax DNA isolation protocol satisfying these criteria and successfully sequenced the extracted nuclear DNA on the ONT platform. The resulting volume of long reads was adequate to assemble a contiguous *L. usitatissimum* line 3896 genome. Due to the reached quality, the genome can be feasibly upgraded to a contiguous chromosome assembly. Therefore, our protocol can be used for obtaining quality flax assemblies using the affordable ONT platform. Our work lays a solid foundation for future research on flax genome structure and emphasizes the importance of selecting the appropriate methodology in plant studies.

## Figures and Tables

**Table 1:** QUAST and BUSCO statistics for the raw genome assemblies of *L. usitatissimum* line 3896 (computed relative to the CDC Bethune assembly and independently).

Assembler	QUAST					BUSCO			QUAST (Reference = CDC Bethune, GCA_000224295.1)		
	Length, Mb	Contigs	N50, Mb	L50	GC, %	C, %	D, %	F, %	Genome Fraction, %	Duplication Ratio	Genomic Features
Canu	447.4	1728	6.2	26	38.8	93.3	59.8	0.8	93.9	1.11	8123 + 20,506
Flye	345.1	7720	0.3	179	39.3	91.8	48.0	1.8	91.5	1.20	7997 + 20,858
Flye (from Canu-corrected reads)	335.9	1571	5.8	21	39.1	93.8	61.8	0.9	93.9	1.06	8219 + 20,309
Miniasm	337.6	1104	0.6	108	39.0	21.7	20.7	11.2	57.8	1.07	1957 + 22,130
NextDenovo	289.5	248	3.1	26	39.4	91.1	44.5	1.3	83.3	1.05	7117 + 18,732
Raven	269.3	1722	0.2	328	39.4	89.7	29.8	1.5	72.4	1.06	4860 + 19,855
Shasta	372.5	6952	1.5	67	38.7	93.2	57.9	0.8	91.0	1.04	7212 + 20,962
SMARTdenovo	163.0	110	2.8	21	39.2	62.9	16.6	1.9	48.9	1.03	4027 + 11,827
Wtdbg2	243.7	3678	0.2	229	40.0	74.5	6.9	4.0	61.7	1.10	214 + 2633

Note: N50 is the maximum length for which the subset of contigs of that length or longer covers at least 50% of the assembly. L50 is the number of contigs with a length equal to or greater than N50, i.e., the minimal number of contigs that cover at least 50% of the assembly. BUSCO: C—complete, D—complete and duplicated, F—fragmented benchmarking universal single-copy orthologs (eudicots_odb10). Genomic features is “the number of genomic features (genes, CDS, etc.) in the assembly (complete + partial), based on a provided list of genomic features positions in the reference genome. A feature is ‘partially covered’ if the assembly contains at least 100 bp of this feature but not the whole one” (https://quast.sourceforge.net/docs/manual.html#sec3.1; accessed on 29 September 2022). Genome fraction is “the total number of aligned bases in the reference, divided by the genome size. A base in the reference genome is counted as aligned if at least one contig has at least one alignment to this base” [57].

**Table 2:** QUAST and BUSCO statistics for the polished genome assemblies of *L. usitatissimum* line 3896 (computed relative to the CDC Bethune assembly and independently).

Assembler + (Polisher)	Polisher	QUAST			BUSCO			QUAST (Reference = CDC Bethune, GCA_000224295.1)		
		Length, Mb	Contigs	N50, Mb	C, %	D, %	F, %	Genomic Features	Mismatches per 100 kbp	Indels per 100 kbp
Canu	-	447.4	1728	6.2	93.3	59.8	0.8	8123 + 20,506 part	1210.3	366.4
	Medaka	448.5	1728	6.2	93.8	62.3	0.8	8403 + 20,441 part	1110.3	268.9
	Medaka ×2	448.6	1728	6.2	93.8	62.3	0.7	8448 + 20,437 part	1094.1	264.8
	NextPolish	450.2	1728	6.2	93.0	54.1	1.0	8150 + 20,705 part	1201.9	342.6
	NextPolish ×2	449.6	1728	6.2	93.7	60.3	0.8	8342 + 20,548 part	1163.5	257.4
	Pepper	424.4	1720	6.3	93.7	62.0	0.9	8446 + 20,402 part	1073.4	229.8
	Pepper ×2	410.9	1661	6.4	93.8	62.2	0.9	8472 + 20,404 part	1074.5	213.9
	Racon	447.6	1701	6.2	93.8	61.1	0.7	8349 + 20,479 part	1156.2	253.5
	Racon ×2	446.7	1695	6.2	93.6	61.1	0.7	8384 + 20,499 part	1143.3	247.4
Canu, Pepper	Medaka	425.0	1715	6.3	93.8	62.2	0.9	8508 + 20,382 part	1061.8	214.3
Canu, Pepper ×2		411.3	1661	6.4	93.8	62.5	0.9	8502 + 20,392 part	1061.0	203.4
Canu, Racon		447.9	1701	6.2	93.8	62.2	0.7	8448 + 20,436 part	1122.0	225.3
Canu, Racon ×2		447.1	1695	6.2	93.8	62.3	0.7	8483 + 20,419 part	1115.3	222.5

**Table 3:** QUAST statistics (for an assembly at the contig level), BUSCO statistics, and repeat content (for a final assembly) for the available *L. usitatissimum* and *L. bienne* genome assemblies.

Assembly	Length, Mb	Contigs	N50, Mb	BUSCO			Total Interspersed Repeats, %
				C, %	D, %	F, %	
3896	447.1	1695	6.2	93.8	62.3	0.7	49.3
Atlant (GCA_014858635.1)	361.8	2458	0.4	94.4	63.4	0.7	44.7
CDC Bethune v.1 (GCA_000224295.1)	282.2	48,397	0.02	93.9	60.4	1.3	33.3
CDC Bethune v.2 (GCA_000224295.2)	316.2	24,829	0.02	93.7	57.4	0.9	27.7
longya 10 (GCA_010665275.2)	306.4	4419	0.2	94.4	60.5	0.9	36.0
Heiya 14 (GCA_010665265.1)	303.7	4581	0.3	94.5	62.6	0.9	36.1
YY5 v.2 (https://zenodo.org/record/4872894)	455.0	336	9.6	94.5	63.1	0.7	50.1
*L. bienne* (GCA_010665285.1)	293.6	6369	0.1	93.3	50.4	1.3	36.3

## Data Availability

The generated dataset for this study can be found in NCBI database under the BioProject accession number PRJNA648016.
